# Treating cutaneous leishmaniasis patients in Kabul, Afghanistan: cost-effectiveness of an operational program in a complex emergency setting

**DOI:** 10.1186/1471-2334-7-3

**Published:** 2007-01-30

**Authors:** Richard Reithinger, Paul G Coleman

**Affiliations:** 1 Clinical Trials Area, Westat, Rockville, USA; 2 Department of Infectious and Tropical Diseases, London School of Hygiene and Tropical Medicine, London, UK; 3 Malaria and Leishmaniasis Control Program, HealthNet TPO, Kabul, Afghanistan

## Abstract

**Background:**

Although Kabul city, Afghanistan, is currently the worldwide largest focus of cutaneous leishmaniasis (CL) with an estimated 67,500 cases, donor interest in CL has been comparatively poor because the disease is non-fatal. Since 1998 HealthNet TPO (HNTPO) has implemented leishmaniasis diagnosis and treatment services in Kabul and in 2003 alone 16,390 were treated patients in six health clinics in and around the city. The aim of our study was to calculate the cost-effectiveness for the implemented treatment regimen of CL patients attending HNTPO clinics in the Afghan complex emergency setting.

**Methods:**

Using clinical and cost data from the on-going operational HNTPO program in Kabul, published and unpublished sources, and discussions with researchers, we developed models that included probabilistic sensitivity analysis to calculate ranges for the cost per disability adjusted life year (DALY) averted for implemented CL treatment regimen. We calculated the cost-effectiveness of intralesional and intramuscular administration of the pentavalent antimonial drug sodium stibogluconate, HNTPO's current CL 'standard treatment'.

**Results:**

The cost of the standard treatment was calculated to be US$ 27 (95% C.I. 20 – 36) per patient treated and cured. The cost per DALY averted per patient cured with the standard treatment was estimated to be approximately US$ 1,200 (761 – 1,827).

**Conclusion:**

According to WHO-CHOICE criteria, treatment of CL in Kabul, Afghanistan, is not a cost-effective health intervention. The rationale for treating CL patients in Afghanistan and elsewhere is discussed.

## Background

In terms of global burden of disease the leishmaniases are the third most important vector-borne disease [1]. Kabul city is currently the world-wide largest focus of cutaneous leishmaniasis (CL) with an estimated 67,500 new cases per annum [2]. Due to its potentially disfiguring pathology, i.e. gross cutaneous lesions that appear at the biting site of the insect vector, the disease has a significant social impact on the affected population (e.g. women with lesions are deemed unsuitable for marriage or to raise children) [3]. However, because the disease is non-fatal, interest by donor agencies to prevent and control the disease has been comparatively poor.

The non-governmental organization (NGO) HealthNet TPO (HNTPO; formerly HealthNet International) has been providing leishmaniasis diagnosis and treatment in Kabul since 1995, the date when the first reports of an imminent CL epidemic emerged [4]. HNTPO's current leishmaniasis activities are embedded in a large, nation-wide operational program (*Malaria and Leishmaniasis Control Program, Afghanistan*) and are focusing on CL microscopic diagnosis and treatment, prevention and control (e.g. through the distribution of insecticide-treated nets to active patients and health education).

In the past decade global humanitarian assistance by the international community on complex emergencies has increased to >US$4.5 billion in 2004, of which 8.5% was spent on the provision of health care [5]. Nevertheless, emergencies repeatedly show that there is a shortage of funds, especially for long-term reconstruction efforts [6]. Critics argue that this funding shortfall is because donor agencies are reluctant to support programs with poor records of impact evaluation. The sheer volume of resources spent on humanitarian aid and the chronicity of many emergencies call for more attention to be paid to the issue of 'value for money', i.e. how to best allocate limited resources to maximize health outcomes.

Cost-effectiveness analyses (CEA) have become a tool that can assist priority setting for health interventions in developing countries, not only by measuring the impact of a particular intervention but also in developing targeted strategies as well as comparing different approaches to treatment. Effectiveness of a given health intervention is usually measured in terms of disability adjusted life years (DALY) averted, a measure of health outcome incorporating premature death and morbidity, or disability [7].

Since the massive humanitarian re-construction efforts began after the fall of the Taliban in 2002, Afghanistan is slowly emerging from two decades of civil war. Nevertheless, country health indicators are still among the worst globally [8] with many preventable diseases such as polio, typhoid fever and measles rampant throughout the country [9]. Despite >US$2.2 billion having been spent on the Afghan health sector since 2002 [5], there has -so far- only been one published study evaluating the cost-effectiveness of a specific health intervention, i.e. a latrine revision program implemented by the International Committee of the Red Cross [10].

The aim of the work reported here was two-fold. Our first aim was to estimate the cost-effectiveness of HNTPO's operational leishmaniasis treatment program in Kabul, Afghanistan (i.e. the comparator being no treatment given to CL patients). Our second aim was to discuss potential policy implications of our results for CL treatment in Afghanistan and elsewhere, especially as CEA for CL prevention and control strategies have not been carried before. Additionally, we highlight characteristics associated with CEA of health programs in complex emergency environments.

## Methods

### Study site and leishmaniasis treatment

Following a widely publicized appeal by the World Health Organization (WHO) [11], HNTPO received funds to expand its leishmaniasis activities in Kabul from one clinic to four fixed health centers and one mobile clinic serving five different locations in and around Kabul; these clinics exclusively treat CL patients. In 2003 HNTPO treated a total 16,390 CL patients at these sites, which according to HNTPO and Afghan Ministry of Health (MoH) estimates represented 75% of all CL cases treated in Kabul.

Patients attending HNTPO leishmaniasis treatment centers were treated with the pentavalent antimonial drug sodium stibogluconate (SSG), either given intralesionally (up to five 2 – 5 ml injections given every 5 – 7 days) or intramuscularly (20 mg/kg bodyweight for 21 consecutive days). Pentavalent antimonial drugs (SbV) are the standard WHO-recommended anti-leishmanial treatment in Afghanistan [12] and elsewhere [13].

### Program costs and clinical parameters

The analyses were based on HNTPO clinic (e.g. patient and staff numbers, underlying demographic and clinical patient characteristics), program and cost figures in 2003.

Costs were primarily obtained from one-year HNTPO budgets approved by international donors and corresponding expenditure reports by HNTPO's accounting department. Other costs were obtained from price catalogues and consultations with researchers and program managers. An ingredients approach was used with costs being divided according to standardized methods into staff time, project management, medical, transportation, equipment, communication and administrative overhead costs [14]. All costs were in 2003 US$.

The cost of HNTPO's 'standard treatment' was estimated by multiplying the total number of patients treated in HNTPO clinics in 2003, i.e. 16,390, with the average amount of drug used per patient, the purchase cost of a 30 ml vial of generic SSG (Albert David, Calcutta, India) and non-medical and medical operational costs as incurred in 2003.

Each HNTPO treatment centre is manned by five health staff (i.e. one medical doctor, one patient registrar, three nurses), working a total 18,000 man-hrs per year. In 2003, 83% and 17% of all patients attending HNTPO clinics were treated with intralesional and intramuscular SSG, respectively. From HNTPO's drug store data we calculated that a total 90 ml and 13 ml of SSG are used when treating patients intramuscularly and intralesionally, respectively (i.e. these figures include drug wastage if new SSG bottles had to be opened when treating new patients).

### Outcome measures

Effectiveness was calculated in terms of DALY averted. For non-fatal diseases, the DALY equals the years of healthy life lost due to the disability (i.e. leishmaniasis) [7], which is the product of leishmaniasis incidence (i.e. 16,390), the standardized disability weight for CL (i.e. 0.023) [15] and the duration of the disease. The duration of disease was arbitrarily set to 12 months following discussion with HNTPO clinicians, which is close to the mean duration of disease observed in a large prevalence survey (i.e. 9.1 months) [2]; unfortunately data on the natural course of *L. tropica *infection does not exist. DALY were discounted at 3%, but they were not weighted for age, because there is controversy over the quantitative impact of the age-weighting function used for burden of disease calculations [16].

Data on treatment effectiveness and compliance for different treatment regimens were obtained from a recent randomized, controlled clinical trial in Kabul [17] and from work carried out elsewhere [18-21]. In the absence of better evidence, we assumed a linear relation between treatment compliance and effectiveness in each case, such that zero compliance results in zero effectiveness, and the degree in compliance achieved in the trial resulted in trial decreases in morbidity. If data was not available, we estimated parameter values based on our operational field experience in Afghanistan and discussions with fellow colleagues.

Our cost effectiveness outputs were based on probabilistic sensitivity analyses [22]. DALY averted due to treatment were combined with costs of each intervention. To allow for a high degree of uncertainty and variability surrounding the variables, probability distributions were assigned to model inputs, i.e. published or estimated values of treatment efficacy and compliance. The distributions were modeled according to Monte Carlo simulations (outputs were based on 5,000 iterations) using @risk software (Palisade Corporation, Newfield, NY) in Microsoft Excel (Microsoft Corporation, Seattle, Washington) spreadsheets, where the software generates the distribution of possible cost-effectiveness ratios by recalculating the spreadsheet for each additional iteration, each time using different randomly selected sets of values from the probability distributions of the input variables. This allows cost-effectiveness outputs to be expressed as a probability distribution rather than as a single point estimate. We calculated the mean and range in which 95% of the cost-effectiveness ratio fell (i.e. the cost-effectiveness range) as summary indicators.

HNTPO is a registered NGO in Afghanistan and the operational program activities described in the manuscript had ethical clearance by the Afghan Ministry of Public Health.

## Results

### Program costs

Non-medical and medical costs for HNTPO's leishmaniasis treatment activities are presented in Table 1. Excluding medical costs, program costs as incurred by HNTPO in 2003 were US$ 129,820. Medical costs to cover the 16,390 patients treated at HNTPO clinics in 2003 were US$ 102,224. Including 7% administrative HNTPO overhead costs, estimated operational costs were approximately one quarter million US$.

**Table 1:** One-year non-medical and medical program costs to treat cutaneous leishmaniasis in Kabul, Afghanistan in 2003.

Item	Total Cost (US$)
*Non-Medical Costs*	
Expatriate Staff	38,880.00
National Staff	39,780.00
Transport	10,800.00
Tools and Equipment	7,740.00
Communication	3,120.00
Operational	28,500.00
*Sub-total*	*128,820.00*
	
*Medical Costs*	
Patient record forms [@US$0.1 pp]	1,639.00
Laboratory materials for drug administration [@US$ 0.5 pp visit]	63,265.40
Sodium stibogluconate [@US$ 3 per vial; 12,440 vials used]	37,320.03
*Sub-Total*	*102,224.43*
	
7% Administrative Overhead Cost	16,173.11
	
*Total Program Cost*	*247,217.54*

Listed are costs for all human and operational resources directly involved in leishmaniasis treatment activities as implemented in Kabul by HealthNet TPO (HNTPO). Costs for these resources were derived from proposals approved by donors as well as corresponding expenditures as reported by HNTPO's accounting department. The cost of the sodium stibogluconate is based on patient treatment regimens outlined in Materials and Methods. Abbreviation: Pp, per person. The full cost breakdown is available from the authors.

### Clinical input parameters and DALY

**Figure 1:**
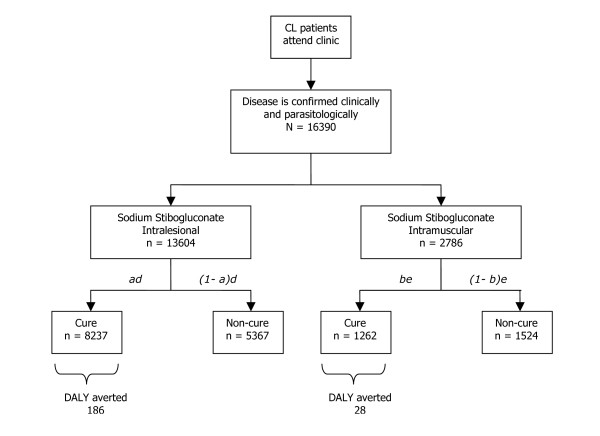
**Flow diagram of cost-effectiveness analysis evaluating different treatment options for cutaneous leishmaniasis in Kabul, Afghanistan**. Represented are the number of patients that were diagnosed, treated and cured in HealthNet TPO's operational leishmaniasis program activities in 2003. Letters represent rates of cure and compliance, as modelled in **Table 2**.

Patient numbers included in our analyses are represented in Figure 1. Modeled clinical parameters are presented in Table 2. The burden of disease of patients attending HNTPO clinics was estimated to be 377 DALY. Because of all patients only a fraction complied with treatment and cured, only 214 DALY were averted due to successful treatment of patients (Figure 1).

**Table 2:** Selected clinical variables for compared treatment protocols.

	*Probability Distribution*		*Simulation Output*	*Reference*
*Treatment Efficacy*				
'Standard Treatment'	Triangular	0.70 Kabul estimate	0.701	Estimated from 17
		0.93 [Maximum]		Estimated from 18 and 20
		0.48 [Minimum]		Estimated from 19 and 21
Intralesional SbV (*a*)	Triangular	0.75 Kabul estimate	0.721	17†
		0.91 [Maximum]		18†
		0.50 [Minimum]		19‡
Intramuscular SbV (*b*)	Triangular	0.45 Kabul estimate	0.604	17
		1.00 [Maximum]		20†
		0.36 [Minimum]		21‡
				
*Treatment Compliance*				
'Standard Treatment'	Triangular	0.74 Kabul estimate	0.824	Estimated from 17
		1.00 [Maximum]		Estimate, not based on data
		0.74 [Minimum]		Estimated from 17
Intralesional SbV (*d*)	Triangular	0.76 Kabul estimate	0.840	17
		1.00 [Maximum]		Estimate, not based on data
		0.76 [Minimum]		17
Intramuscular SbV (*e*)	Triangular	0.62 Kabul estimate	0.750	17
		1.00 [Maximum]		Estimate, not based on data
		0.62 [Minimum]		17
				
*Duration of disease (years)*			1.000	Estimate, not based on data

Parameter distribution was simulated as described using @risk software. The triangular distribution was used to describe clinical parameter distributions. This is standard practice in cost-effectiveness analyses^22 ^to estimate parameter inputs for which the variation is known (minimum and maximum estimates, Kabul estimate), but for which the precise nature of the distribution is not. Parameter estimates were taken from published clinical trials evaluating treatment alternatives for cutaneous leishmaniasis (minimum and maximum estimates) with data from HealthNet TPO's operational program in Kabul (Kabul estimate)^17 ^being our 'best guess' and, hence, mode of the distribution. a, b, d, e refer to parameter estimates used in **Figure 1**. Drug efficacy estimates came from studies evaluating the therapeutic efficacy of pentavalent antimonial drugs (SbV) for cutaneous leishmaniasis, i.e. sodium stibogluconate (†) or meglumine antimoniate (‡)

### Per patient treatment costs

Using the number of observed patient numbers (i.e. 16,390) treated per year, program costs (Table 1), and the HNTPO standard treatment's estimated efficacy and compliance (Table 2), the cost per patient treated and cured in 2003 with the reference treatment was US$ 26.7 (95% CI 19.9 – 35.9).

The cost per DALY averted with HNTPO's standard treatment was estimated at US$ 1180.5 (95% CI: 760.6 – 1826.9).

**Figure 2:**
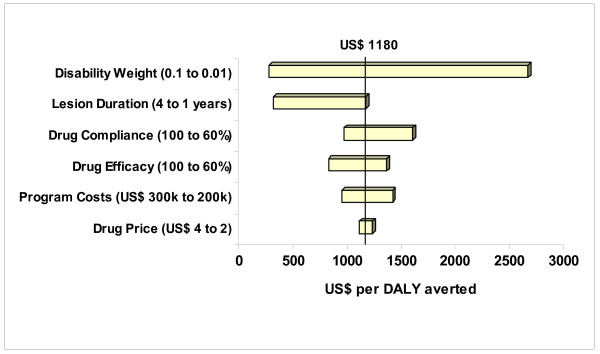
**Tornado diagram of the impact of variation of model input parameters on cost-effectiveness estimates for cutaneous leishmaniasis treatment in Kabul, Afghanistan**. Tornado diagram showing how cost-effectiveness of cutaneous leishmaniasis treatment would change if one of the input parameters would be reduced or increased, as specified.

Figure 2 represents the change in cost per DALY averted when one of the model input parameters is reduced or increased, as specified.

## Discussion

We estimated the cost-effectiveness of an operational CL treatment program in Kabul, Afghanistan. This was done for internal program evaluation purposes (i.e. to assess program impact), but also to yield comparative data that would be of use for the local MoH and other health sector NGOs and stakeholders. Such data and exercises are rare [10], despite repeated calls for health interventions in complex emergencies to be evidence-based and assessed for their cost-effectiveness [23-25]. In our approach we extrapolate results from published clinical research trials to a defined operational setting, estimating the cost-effectiveness of the currently used combination of intralesional and intramuscular SSG in Kabul, Afghanistan (i.e. 'standard treatment'). We express results as realistic ranges rather than simple point estimates, thereby including the uncertainty surrounding used input parameters.

### The cost-effectiveness of current leishmaniasis treatment in Kabul, Afghanistan and its comparison to alternative treatment protocols

Using chosen input parameters, we estimate that the current cost per patient treated and cured in HNTPO's operational program in Kabul is US$ 27. The cost per patient treated and cured with SSG we report here was lower than expected, and is much lower than the SbV treatment costs reported in other CL-endemic countries, notably US$ 280 in Guatemala [26], US$ 300 in Peru [27], and US$ 5500 in the USA. (Aronson, *pers. comm*.). This outcome is due to the difference in drug purchasing price (e.g. use of generic versus branded SbV [Pentostam^® ^or Glucantime^®^]), therapy protocol (e.g. intralesional versus intramuscular versus intravenous administration of SbV), labor costs (e.g. Afghan versus American health staff), and patient care (i.e. treatment is on an out-patient basis in Afghanistan versus an in-patient basis in the USA.).

For the first time, the cost-effectiveness of CL treatment is evaluated. Using parameter estimates listed in Table 1 we estimate that the cost-effectiveness of CL treatment in Kabul is approximately US$ 1,200 per DALY averted.

### Policy implications for cutaneous leishmaniasis treatment in Kabul and elsewhere

According to WHO-CHOICE criteria health interventions are 'very cost-effective' and 'cost-effective' if within one and three times a country's per capita GDP, respectively [28]. The per capita GDP for Afghanistan was US$ 165 in 2004 and US$ 232 in 2006 [29]. Hence, with US$ 1,200 per DALY averted CL treatment in Kabul is not a 'cost-effective' health intervention, and based on above criteria it cannot be argued that it is a justified health expenditure.

Although the methods to estimate an intervention's cost-effectiveness may not be strictly comparable, we can set our figures into context, with other CEA having found the cost-effectiveness of supplemental tetanus immunization in Pakistan to be US$ 2 – 6 per DALY averted [30], melarsoprol treatment for late stage African trypanosomiasis in Uganda US$ 8 [31], SSG treatment for fatal visceral leishmaniasis US$18 [32], ivermectin distribution for onchocerciasis control US$ 14 – 30 [33], malaria chloroquine chemoprophylaxis US$ 14 – 93 [22], integrated water supply and sanitation US$ 20 – 1,152 [34], and the mass-treatment of trachoma patients with azythromycin US$ 9,000 – 65,000 [35].

In many leishmaniasis-endemic countries, the standard recommended treatment for CL is the intramuscular administration with SbV [13]. Had patients been treated with intramuscular SSG only, 6,492 patients could have been treated due to available staff man-hrs (i.e. due to the higher number of patient-clinic visits required when following this regimen), which would have yielded an estimate of US$ 3,718 per DALY averted (data not shown). Clearly, in those countries increased use of intralesional SbV as treatment regimen as in HNTPO's operational program would increase the cost-effectiveness of anti-leishmanial treatment dramatically.

However, whilst our analyses appear to demonstrate that CL treatment in Kabul is not cost-effective according to WHO-CHOICE criteria, several caveats of our analysis have to be highlighted. First, as with any CEA, some of the model input parameters are debatable. For example, we arbitrarily chose a lesion duration of 12 months even though many patients can have lesions of longer duration [2]; undeniably, by doing so, we may have underestimated true lesion duration due to *L. tropica *infection. As shown in Figure 2, a longer lesion duration can have a significant impact on the cost-effectiveness ratio. Unfortunately, we are unaware of any published data on the natural course of *L. tropica *infections, and used data represents our best estimate. Similarly, data used for clinical efficacy in our evaluation has been extrapolated from studies on two SbV, i.e. meglumine antimoniate and SSG. No data has been published demonstrating that both drugs have varying degree of efficacy in treating CL and used data, again, represents our best estimate of minimum and maximum values of the treatment efficacy distribution.

Second, as has been the case for schistosomiasis [36] and other communicable diseases [37], the disability weighting for CL as set by the Global Burden of Disease study [7,15] is debatable and should be re-evaluated. CL can cause significant scarring as well as chronic pathology, which in some cultural contexts such as Afghanistan can lead to severe ostracism of the affected population [3], especially when scars are located on the face. In such instances, DALY should not only be estimated for the duration of active leishmaniasis lesions, but for the duration of scars as well. Inclusion of disability incurred due to leishmaniasis scars in our DALY estimates would have significantly altered our estimated cost-effectiveness range. Thus, a disability weight similar to a cleft lip (i.e. 0.049), Bancroftian filariasis (i.e. 0.106) or debilitating leprosy (i.e. 0.152) [15], the cost-effectiveness of CL treatment in Kabul would have been US$ 541, US$ 250, and US$ 174 per DALY averted, respectively (see also Figure 2).

Third, once patients cure from leishmaniasis they tend to be immune to re-infection [38]. Thus, unlike other infectious diseases such as dengue or malaria there will be no recurrent DALY or treatment costs per person.

Fourth, CL in Kabul is transmitted anthroponotically with active cases presumed to be highly infectious to sandfly vectors [4]. Although epidemiological data investigating the impact of mass treatment on anthroponotic CL transmission does not exist, it is likely that treating and curing leishmaniasis patients will reduce the size of the reservoir and therefore avert further new cases (i.e. DALY). How many cases would be prevented by treating one active case is difficult to quantify, but it would reduce the cost-effectiveness ratio.

Fifth, it is clear that our study only applies to the given, local conditions in Kabul, with analyses being based on chosen input parameters and care should be given not to generalize the conclusions. Cost figures were primarily based on an operational program implemented locally and are likely to vary when compared to programs elsewhere.

In future work we plan to carry out a range of sensitivity analyses to investigate the effect of a range of cost and clinical parameters on estimated treatment cost-effectiveness. For example, cost-effectiveness ratios would have been higher had estimates been based on fewer patients attending the clinics.

Whilst it is clear that not all patients can be treated with intralesional SbV (e.g. due to the painful route of drug administration patients with lesions close to the eyes or lips tend to be given intramuscular SbV instead) our results show that costs to treat CL patients could be substantially reduced were a treatment strategy implemented that would largely adopt the localized treatment of CL lesions.

### Difficulties associated with cost-effectiveness analyses in complex emergencies

It is surprising that cost-effectiveness analyses of health interventions in complex emergencies are very scarce [10,32], highlighting a major gap in the evidence-based implementation of humanitarian programs [23]. Whilst it is true that there are many logistical constraints in collecting reliable financial and operational data in complex emergencies (e.g. due to the absence of established health information systems) [39], health interventions in these settings are often vertical with program-allocated budgets allowing for easy data collection. CEA should enable local stakeholders to set health intervention priorities, use results as leverage to obtain funding as well as make implementing partners more accountable in front of donor organizations. The drawback is that the CEA will be based on local circumstances, which can vary considerably between and within emergencies. However, it is clear that within a local context, CEA will assist priority setting of health interventions, whether for comparative purposes between different diseases or a single disease [40].

Thus, for example, despite the findings reported here, HNTPO's treatment activities currently implemented in Kabul are still substantially more cost-effective than a household latrine revision intervention to reduce diarrheal disease [10].

## Conclusion

Because CL is not fatal, it is largely ignored by international aid and donor agencies, and it has become one of the so-called 'neglected diseases'. Our results do little in refuting this belief, as we demonstrate that the standard, WHO-recommended treatment of CL in Afghanistan is -according to WHO-CHOICE criteria- not cost-effective. Efforts should be made to develop and standardize short systemic treatment regimens for CL as well as to develop regimens of localized treatment alternatives (e.g. intralesional SSG) which may improve cost-effectiveness.

## Competing interests

RR has been a consultant to Thermosurgery Technologies Inc., received conference travel funds from Zentaris AG and was provided free miltefosine by Zentaris AG to carry out a clinical trial on its efficacy in Afghanistan. Paul Coleman has no conflict of interest declared.

## Authors' contributions

RR initiated the research, collected operational data, reviewed the literature, and did the analyses. PG designed models and did the analyses. Both authors contributed to the writing of the paper, read and approved the final manuscript.

## Pre-publication history

The pre-publication history for this paper can be accessed here:



## References

[B1] World Health Organization (2004). The World Health Report. Changing History.

[B2] Reithinger R, Mohsen M, Aadil K, Sidiqi M, Erasmus P, Coleman PG (2003). Anthroponotic cutaneous leishmaniasis, Kabul, Afghanistan. Emerg Infect Dis.

[B3] Reithinger R, Aadil K, Kolaczinski J, Mohsen M, Hami S (2005). Social impact of leishmaniasis, Afghanistan. Emerg Infect Dis.

[B4] Ashford RW, Kohestany KA, Karimzad MA (1992). Cutaneous leishmaniasis in Kabul: observations on a 'prolonged epidemic'. Ann Trop Med Parasitol.

[B5] UN Office for Coordination of Humanitarian Affairs Financial Tracking Service – Tracking global humanitarian aid flows. http://ocha.unog.ch/fts/index.aspx.

[B6] Walker P, Wisner B, Leaning J, Minear L (2005). Smoke and mirrors: deficiencies in disaster funding. BMJ.

[B7] Murray CJL, Lopez AD (1996). The global burden of disease: a comprehensive assessment of mortality and disability from diseases, injuries and risk factors in 1990 and projected to 2020.

[B8] United Nations Development Program (2004). Afghanistan National Human Development Report 2004: Security with a human face – challenges and responsibilities.

[B9] Waldmann R, Hanif H (2002). Afghanistan Research Evaluation Unit. Issues Paper Series. The Public Health System in Afghanistan – Current Issues.

[B10] Meddings DR, Ronald LA, Marion S, Pinera JF, Oppliger A (2004). Cost-effectiveness of a latrine revision program in Kabul, Afghanistan. Bull WHO.

[B11] Anonymous (2004). WHO action in Afghanistan aims to control debilitating leishmaniasis. Wkly Epidemiol Rec.

[B12] Reyburn H (2000). A Guide to the treatment of cutaneous leishmaniasis.

[B13] Herwaldt BL (1999). Leishmaniasis. Lancet.

[B14] Johns B, Baltussen R, Hutubessy R (2003). Program costs in the economic evaluation of program costs. Cost Effectiveness and Resource Allocation.

[B15] Lopez AD, Mathers CD, Ezzati M, Jamison DT, Murray CJL (2006). Global Burden if Disease and Risk Factors.

[B16] Barendregt JJ, Bonneux L, Van der Maas PJ (1996). DALYs: the age-weights on balance. Bull WHO.

[B17] Reithinger R, Mohsen M, Wahid M, Bismullah M, Quinnell RJ, Davies CR, Kolaczinski J, David JR (2005). Efficacy of thermotherapy to treat cutaneous leishmaniasis caused by *Leishmania tropica *in Kabul, Afghanistan: a randomized, controlled trial. Clin Infect Dis.

[B18] Tallab TM, Bahamdam KA, Mirdad S, Johargi H, Mourad MM, Ibrahim K, el Sherbini AH, Karkashan E, Khare AK, Jamal A (1996). Cutaneous leishmaniasis: schedules for intralesional treatment with sodium stibogluconate. Int J Dermatol.

[B19] Asilian A, Sadeghinia A, Faghihi G, Momeni A, Amini Harandi A (2003). The efficacy of treatment with intralesional meglumine antimoniate alone, compared with that of cryotherapy combined with the meglumine antimoniate or intralesional sodium stibogluconate, in the treatment of cutaneous leishmaniasis. Ann Trop Med Parasitol.

[B20] Ballou WR, McClain JB, Gordon DM, Shanks GD, Andujar J, Berman JD, Chulay JD (1987). Safety and efficacy of high-dose sodium stibogluconate therapy of American cutaneous leishmaniasis. Lancet.

[B21] Martinez S, Marr JJ (1992). Allopurinol in the treatment of American cutaneous leishmaniasis. N Engl J Med.

[B22] Goodman CA, Coleman PG, Mills AJ (1999). Cost-effectiveness of malaria control in sub-Saharan Africa. Lancet.

[B23] Banatvala N, Zwi AB (2000). Conflict and health: public health and humanitarian interventions: developing the evidence base. BMJ.

[B24] Duffield A, Reid G, Shoham J, Walker D (2005). Evidence base for interventions in complex emergencies. Lancet.

[B25] Connolly MA, Gayer M, Ryan MJ, Salama P, Spiegel P, Heymann DL (2004). Communicable disease in complex emergencies: impact and challenges. Lancet.

[B26] Arana BA, Mendoza CE, Rizzo NR, Kroeger A (2001). Randomized, controlled, double-blind trial of topical treatment of cutaneous leishmaniasis with paromomycin plus methylbenzethonium chloride ointment in Guatemala. Am J Trop Med Hyg.

[B27] Guthmann JP, Arlt D, Garcia LM, Rosales M, de Jesus Sanchez J, Alvarez E, Lonlas S, Conte M, Bertoletti G, Fournier C, Huari R, Torreele E, Llanos-Cuentas A (2005). Control of mucocutaneous leishmaniasis, a neglected disease: results of a control program in Satipo Province, Peru. Trop Med Int Health.

[B28] Tan-Torres Edejer T, Baltussen R, Adam T, Hutubessy R, Acharya A, Evans DB, Murray CJL (2003). WHO Guide to Cost-effectiveness Analysis.

[B29] US Department of State Background Note Afghanistan Profile. http://www.state.gov/r/pa/ei/bgn/5380.htm.

[B30] Griffiths UK, Wolfson LJ, Quddus A, Younus M, Hafiz RA (2004). Incremental cost-effectiveness of supplementary immunization activities to prevent neonatal tetanus in Pakistan. Bull WHO.

[B31] Politi C, Carrin G, Evans D, Kuzoe FA, Cattand PD (1995). Cost-effectiveness analysis of alternative treatments of African *gambiense *trypanosomiasis in Uganda. Health Econ.

[B32] Griekspoor A, Sondorp E, Vos T (1999). Cost-effectiveness analysis of humanitarian relief interventions: visceral leishmaniasis treatment in the Sudan. Health Policy and Planning.

[B33] Waters HR, Rehwinkel JA, Burnham G (2004). Economic evaluation of Mectizan distribution. Trop Med Int Healt.

[B34] Varley RC, Tarvid J, Chao DN (1998). A reassessment of the cost-effectiveness of water and sanitation interventions in programs for controlling childhood diarrhea. Bull WHO.

[B35] Baltussen RM, Sylla M, Frick KD, Mariotti SP (2005). Cost-effectiveness of trachoma control in seven world regions. Ophthalmic Epidemiol.

[B36] King CH, Dickman K, Tisch DJ (2005). Reassessment of the cost of chronic helminthic infection: a meta-analysis of disability-related outcomes in endemic schistosomiasis. Lancet.

[B37] Arnesen T, Kapiriri L (2004). Can the value choices in DALYs influence global priority-setting?. Health Policy.

[B38] Handman E (2001). Leishmaniasis: current status of vaccine development. Clin Microbiol Rev.

[B39] Thieren M (2005). Health information systems in humanitarian emergencies. Bull WHO.

[B40] Kapiriri L, Arnesen T, Norheim OF (2004). Is cost-effectiveness analysis preferred to severity of disease as the main guiding principle in priority setting in resource poor settings? The case of Uganda. Cost Eff Resour Alloc.

